# Learning-based object's stiffness and shape estimation with confidence level in multi-fingered hand grasping

**DOI:** 10.3389/fnbot.2024.1466630

**Published:** 2024-11-19

**Authors:** Kyo Kutsuzawa, Minami Matsumoto, Dai Owaki, Mitsuhiro Hayashibe

**Affiliations:** Neuro-Robotics Laboratory, Department of Robotics, Graduate School of Engineering, Tohoku University, Sendai, Japan

**Keywords:** robotic hand, grasping, stiffness estimation, shape estimation, probabilistic inference, deep learning, proprioception

## Abstract

**Introduction:**

When humans grasp an object, they are capable of recognizing its characteristics, such as its stiffness and shape, through the sensation of their hands. They can also determine their level of confidence in the estimated object properties. In this study, we developed a method for multi-fingered hands to estimate both physical and geometric properties, such as the stiffness and shape of an object. Their confidence levels were measured using proprioceptive signals, such as joint angles and velocity.

**Method:**

We have developed a learning framework based on probabilistic inference that does not necessitate hyperparameters to maintain equilibrium between the estimation of diverse types of properties. Using this framework, we have implemented recurrent neural networks that estimate the stiffness and shape of grasped objects with their uncertainty in real time.

**Results:**

We demonstrated that the trained neural networks are capable of representing the confidence level of estimation that includes the degree of uncertainty and task difficulty in the form of variance and entropy.

**Discussion:**

We believe that this approach will contribute to reliable state estimation. Our approach would also be able to combine with flexible object manipulation and probabilistic inference-based decision making.

## 1 Introduction

Human beings possess a remarkable capacity to discern physical characteristics of grasped objects, such as stiffness and shape, from the sensory signals of the hand with a high degree of freedom. Furthermore, but they are also able to recognize their confidence in the estimated object properties, which they use for manipulation and further exploration. For example, humans are able to recognize the shape of an object by touching it. When the shape is unclear at the first touch, they recognize it and touch the object again to make sure where to grasp. When the grasped object is likely to be soft and fragile, humans would grasp it conservatively based on the degree of uncertainty in the rigidity. These abilities are essential to realize robotic hands that are capable of performing stable grasping and manipulation.

Object properties can be classified into two distinct types of categories: geometric properties and physical properties (Wang et al., 2020). The geometric properties include the position, pose, shape, etc., whereas the physical properties include the mass, stiffness, texture, etc. Estimating both types of properties is essential for proper object manipulation. For example, the geometric properties provide the necessary posture of the hand necessary to grasp the object and maintain its form closure, which is crucial for stable grasping, while the physical properties determine the maximum amount of grasping force the robot can exert without breaking the object. Robots need to know those properties from sensory information unless those properties were given in advance. However, putting multiple various sensors on the hand can increase the cost. Thus, it is preferable to estimate the object properties from proprioceptive information, such as finger joint angles, which can be measured in most cases for joint control.

There are studies regarding the estimation of geometric and physical properties with robotic hands (Wang et al., 2020; Spiers et al., 2016; Gao et al., 2016). However, in contrast to estimation of geometric properties. It has been widely researched that the estimation of physical properties or combining them with geometric-property estimation is not fully researched. There exist numerous methods for estimating physical-property. The estimation of stiffness necessitates the utilization of additional sensors for force measurements (Kicki et al., 2019; Spiers et al., 2016), resulting in an increase in costs and mechanical complexity. Furthermore, it has been observed that stiffness estimation is typically performed solely once at the terminal time step (Bednarek et al., 2021). For adaptive grasping and dexterous manipulation, robotic hands need to estimate both types of object properties in real time in order to adjust grasping force.

When a robotic hand estimates object properties from proprioception in real time, it is important to handle uncertainty as this task requires to estimate from limited information. For example, it is impossible to estimate the objects' properties of objects before contact, which results in the *epistemic* uncertainty, which is caused by lack of knowledge. Furthermore, observation noises in sensory signals degrade the quality of estimation, resulting in the *aleatoric* uncertainty, which is due to randomness. Without estimating those uncertainties, robots would make incorrect decisions based on unreliable estimates that could have been avoided with further exploration and conservative actions. Another issue that arises when estimating object is the necessity of weight parameters in the loss function to balance the scales of the multiple properties, which that is a time-consuming process.

To address the above issues, this study introduces a method to estimate both geometric and physical properties using confidence factors, which measure uncertainty. We develop design a learning framework based on probabilistic inference and apply the method to neural networks with a robotic hand without tactile sensors in simulation. In the framework, we utilized a loss function without hyper-parameters and a time-series chunking technique that could improve learning stability. Neural networks are implemented to generate the variance of the estimated stiffness, which value can be regarded as the confidence level of estimation. Although neural networks outputting variance are not novel (Nix and Weigend, 1994), we apply this approach to object-property estimation with a multi-fingered robotic hand and demonstrate its effectiveness to this task. Contributions from of this study are listed below.

1. We have developed a framework that enables for robotic hands to assess stiffness and shape of an object, incorporating their uncertainty.
2. We designed a loss function without hyperparameters in order to balance the scales between different properties.
3. We demonstrate that trained neural networks are capable of estimating the stiffness and the shape by utilizing proprioceptive signals, while also estimating the confidence level of estimation, taking into account and task difficulty, such as variance and entropy.

## 2 Materials and methods

### 2.1 Overview

We consider a situation where a robotic hand grasps an object with pre-defined control commands and estimates the object's properties, particularly its stiffness and the shape. The robot hand is capable of measuring joint angles and joint angular velocity, but it does not have visual sensors, tactile sensors, or force sensors. We finally develop neural networks that generate estimates of the object's properties sequentially.

We define mathematical symbols as follows. Let the stiffness be expressed as a scalar value *k*>0, and let the shape be expressed as a discrete label $s\in S≜\left\{S_{1},\ldots ,S_{C}\right\}$, where *C* denotes the number of classes. Furthermore, it is recommended that the joint angles, joint angular velocities, and joint angle commands be designated as ***q***∈ℝ^*D*^, $\overset{\cdot }{q}\in {\mathbb{R}}^{D}$, and ***q***^cmd^∈ℝ^*D*^, respectively, where *D* denoting the degrees of freedom (DoF). We occasionally refer to observations as $y≜{\left[q,\overset{\cdot }{q},q^{\text{cmd}}\right]}^{⊤}\in {\mathbb{R}}^{3D}$ for simplicity.

### 2.2 Training strategy

In many studies, the root mean square errors (RMSEs) are used to estimate continuous values. However, there are a few that make the object property estimation difficult:

1. It is impossible in principle to estimate the properties of an object properties before contact with the object, resulting in the epistemic uncertainty. RMSEs are unable to handle such kind of uncertainty.
2. As neural networks are capable of estimating multiple types of properties with varying units and different representations (e.g., continuous values or discrete labels), such as weight constants are typically required to balance the estimation errors among various types of properties. The cost of designing weight constants increases with the increase in the number of properties.

To address the above issues, we designed the estimation task as a probabilistic inference. Probabilistic inference can naturally express the uncertainty of the object's properties that happens before touch. Furthermore, by considering the task as a likelihood maximization problem, it can be transformed into probabilistic inference of a single joint distribution of multiple properties, which does not have weight constants.

Concretely, the object property estimation task is formulated as the following optimization problem:


(1) $$\begin{array}{l} \underset{\theta_{n}^{k},\theta_{n}^{s}}{\mathrm{maximize}}\overset{N-1}{\underset{n=0}{\prod }}p\left(k_{n},s_{n}|\theta_{n}^{k},\theta_{n}^{s}\right). \end{array}$$

Here, *N* and *n* indicate the dataset size and the index of samples in the dataset. $\theta_{n}^{k}$ and $\theta_{n}^{s}$ indicate the parameters of the probabilistic distributions of the stiffness and the shape, respectively. $\theta_{n}^{k}$ and $\theta_{n}^{s}$ can be regarded as estimation results of *k*_*n*_ and *s*_*n*_ in a statistic way. We then consider its negative log-likelihood as follows:


(2) $$\begin{array}{l} -\log\overset{N-1}{\underset{n=0}{\prod }}p\left(k_{n},s_{n}|\theta_{n}^{k},\theta_{n}^{s}\right)=\overset{N-1}{\underset{n=0}{\sum }}-\log p\left(k_{n},s_{n}|\theta_{n}^{k},\theta_{n}^{s}\right). \end{array}$$

Thus, the optimization problem can be equivalently converted as follows:


(3) $$\begin{array}{l} \underset{\theta_{n}^{k},\theta_{n}^{s}}{\mathrm{minimize}}\overset{N-1}{\underset{n=0}{\sum }}\left[-\log p\left(k_{n},s_{n}|\theta_{n}^{k},\theta_{n}^{s}\right)\right]. \end{array}$$

We assume each negative log-likelihood term can be decomposed as follows:


(4) $$\begin{array}{l} -\log p\left(k_{n},s_{n}|\theta_{n}^{k},\theta_{n}^{s}\right)=-\log\left[p\left(k_{n}|\theta_{n}^{k}\right)p\left(s_{n}|\theta_{n}^{s}\right)\right]\\ \;=-\log p\left(k_{n}|\theta_{n}^{k}\right)-\log p\left(s_{n}|\theta_{n}^{s}\right) \end{array}$$

We also model the probabilistic distribution of the stiffness as a Gaussian distribution as follows:


(5) $$\begin{array}{l} -\log p\left(k_{n}|\theta_{n}^{k}\right)=-\log p\left(k_{n}|\mu_{n},\sigma_{n}^{2}\right)\\ \;=-\log\left(\frac{1}{\sqrt{2\pi \sigma_{n}^{2}}}\exp\left[-\frac{{\left(k_{n}-\mu_{n}\right)}^{2}}{2\sigma_{n}^{2}}\right]\right)\\ \;=\frac{{\left(k_{n}-\mu_{n}\right)}^{2}}{2\sigma_{n}^{2}}+\log\sigma_{n}+\frac{1}{2}\log\left(2\pi \right) \end{array}$$

Here, the parameter is expressed as $\theta_{n}^{k}≜\left(\mu_{n},\sigma_{n}^{2}\right)$, where μ_*n*_ and $\sigma_{n}^{2}$ indicate the mean and the variance. On the other hand, the probabilistic distribution of the shape is modeled as a discrete distribution obtained through the softmax function as follows:


(6) $$\begin{array}{l} -\log p\left(s_{n}=S_{c}|\theta_{n}^{s}\right)=-\log p\left(s_{n}=S_{c}|z_{n}^{\left(1\right)},\ldots ,z_{n}^{\left(C\right)}\right)\\ \;=-\log\frac{\exp z_{n}^{\left(c\right)}}{\sum_{c^{\prime }=1}^{C}\exp z_{n}^{\left(c^{\prime }\right)}} \end{array}$$

Here, the parameter is expressed as $\theta_{n}^{s}≜\left(z_{n}^{\left(1\right)},\ldots ,z_{n}^{\left(C\right)}\right)$, where $z_{n}^{\left(c\right)}\in \mathbb{R}$ for all *c* = 1, …, *C*. The above equation corresponds to the cross-entropy. Hereinafter, we use $z_{n}≜{\left[z_{n}^{\left(1\right)},\ldots ,z_{n}^{\left(C\right)}\right]}^{⊤}$ and $\text{CE}\left(s_{n},z_{n}\right)≜-\log p\left(s_{n}|\theta_{n}^{s}\right)$.

Finally, the optimization problem is converted as follows:


(7) $$\begin{array}{l} \underset{\mu_{n},\sigma_{n},z_{n}}{\mathrm{minimize}}\overset{N-1}{\underset{n=0}{\sum }}\left[\frac{{\left(k_{n}-\mu_{n}\right)}^{2}}{2\sigma_{n}^{2}}+\log\sigma_{n}+\text{CE}\left(s_{n},z_{n}\right)\right]. \end{array}$$

This can be computed by minimizing the following learning loss:


(8) $$\begin{array}{l} L≜\frac{1}{N}\overset{N-1}{\underset{n=0}{\sum }}\left[\frac{{\left(k_{n}-\mu_{n}\right)}^{2}}{2\sigma_{n}^{2}}+\log\sigma_{n}+\text{CE}\left(s_{n},z_{n}\right)\right]. \end{array}$$

It is noteworthy that the learning loss *L* lacks any hyper-parameters, such as weight constants. Instead, σ_*n*_ behaves as a weight balancing the RMSE of the stiffness ${\left(k_{n}-\mu_{n}\right)}^{2}$ and the classification errors of the shape CE(*s*_*n*_, ***z***_*n*_). Unlike a constant weight, σ_*n*_ itself is to be optimized by the term of logσ_*n*_, resulting in a statistically optimal value.

### 2.3 Estimation with neural networks

We develop a neural network architecture that estimates parameters such as $\theta_{n}^{k}$ and $\theta_{n}^{s}$ from a time series of observations. A simple approach is to use recurrent neural networks that receive observations *y* for each time step. However, it may lead to too deep layers in time, which may result in unstable learning, high computational cost, and slow inference. Therefore, we treat a time series of raw observations with a high sampling rate as a time series of chunks with a low sampling rate (Kutsuzawa et al., 2017, 2018). Similar techniques have also been employed in a Transformer-based model for robotic imitation learning (Zhao et al., 2023). Concretely, we transformed a time series (***y***[0], ***y***[1], …, ***y***[*t*], …, ***y***[*T*−1]) into (***Υ***[0], ***Υ***[1], …, ***Υ***[λ], …, ***Υ***[Λ−1]), where


(9) $$\begin{array}{l} \Upsilon \left[\lambda \right]≜{\left[y^{⊤}\left[\lambda K\right],y^{⊤}\left[\lambda K+1\right],\ldots ,y^{⊤}\left[\left(\lambda +1\right)K-1\right]\right]}^{⊤}\in {\mathbb{R}}^{3DK}. \end{array}$$

**Figure 1:**
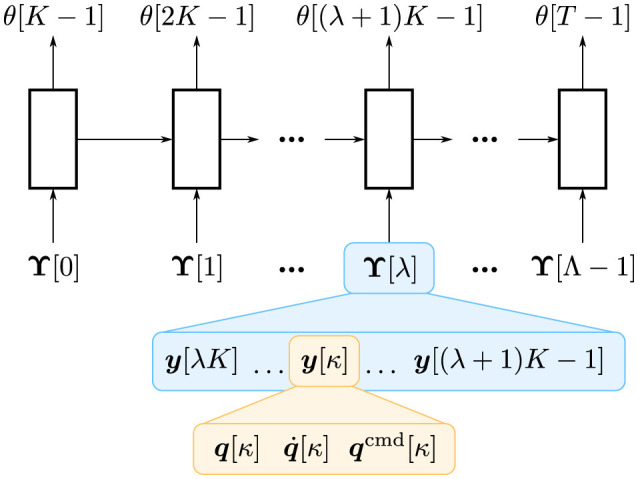
Overview of time-series chunking. A recurrent neural network, illustrated as blocks, receives ***Υ***[λ] that consists of *K* observations: (***y***[λ*K*], ***y***[λ*K*+1], …, ***y***[(λ+1)*K*−1]). θ[*t*] indicates the estimated parameters of the stiffness and the shape at the *t*-th time step, i.e., θ[*t*] = (θ^*k*^[*t*], θ^*s*^[*t*]).

Here, *K*∈ℕ indicates the chunk size. Finally, the time-series length of *T* can be reduced to a shorter length $\Lambda =\frac{T}{K}\in \mathbb{N}$, where ⌈•⌉ indicates the ceiling function. This technique can reduce the time-series length approximately *K* times shorter, making learning more stable with a lower computational cost. Although it also reduces the estimation frequency, it does not matter in many cases as estimation usually does not require a high update frequency. Although chunking may limit the representation ability of the model, it would be better than down-sampling, which is similar to chunking but decimates the data samples. Thus, it is anticipated that this chunking technique yields more advantages than disadvantages. This technique is graphically shown in Figure 1.

A neural network generates the values of, μ, logσ^2^, and ***z***. Here, we use logσ^2^ instead of σ or σ^2^ as a primitive term because logσ^2^ can take −∞ to ∞, making it more manageable for a linear-combination layer. Therefore, in practice, the learning loss defined in Equation 8 is transformed as follows:


(10) $$\begin{array}{l} L=\frac{1}{N}\overset{N-1}{\underset{n=0}{\sum }}\left[\frac{{\left(k_{n}-\mu_{n}\right)}^{2}}{2\exp\left(\log\sigma_{n}^{2}\right)}+\frac{1}{2}\log\sigma_{n}^{2}+\text{CE}\left(s_{n},z_{n}\right)\right]. \end{array}$$

### 2.4 Evaluation setup

**Figure 2:**
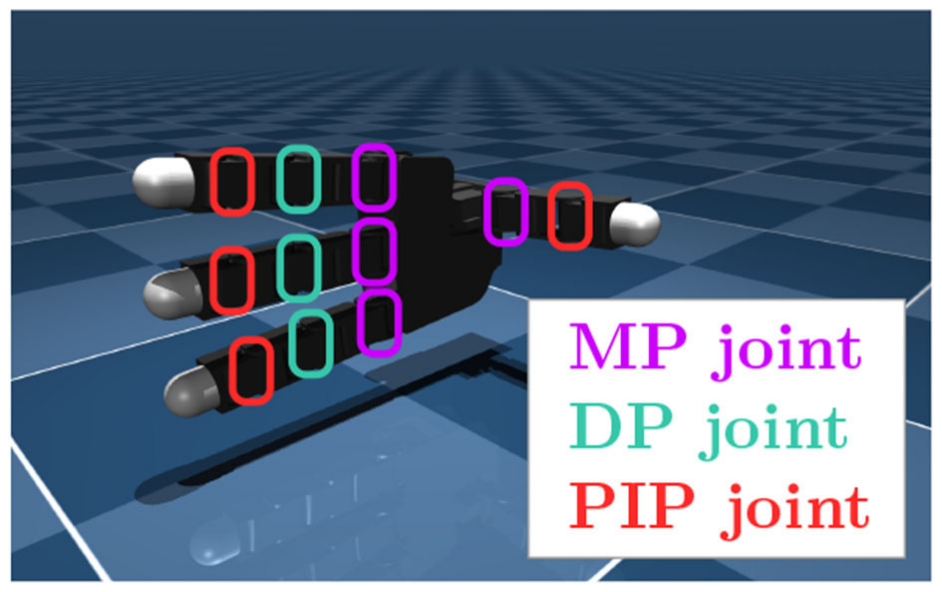
Allegro Hand and its joint definitions.

We used a MuJoCo (Todorov et al., 2012) implementation of Allegro Hand, implemented by Zakka et al. (2022), as shown in Figure 2. We controlled flexion motion at the joints; 11 degrees of freedom were obtained. The entire process is controlled.

**Figure 3:**
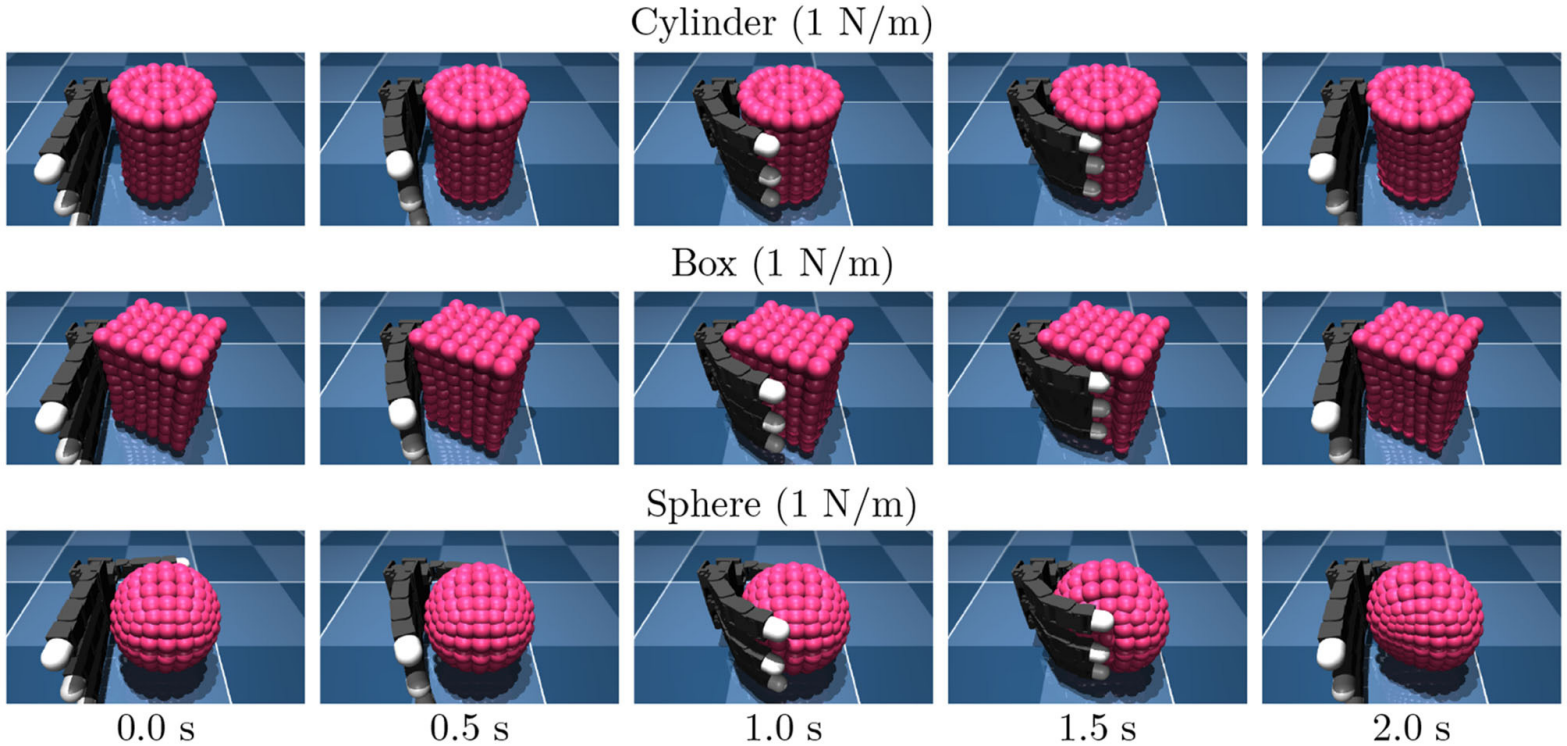
Snapshots of the training data.

We employed three distinct categories of target objects: cylinder-shaped objects, box-shaped objects, namely sphere-shaped objects, as shown in Figure 3. An object was modeled as a composite of small capsule elements connected by springs each other. The object stiffness was determined by specifying the spring stiffness connecting the elements. Objects were fixed to the space to avoid falling out of the hand. Note that this study focuses on the object property estimation.

**Figure 4:**
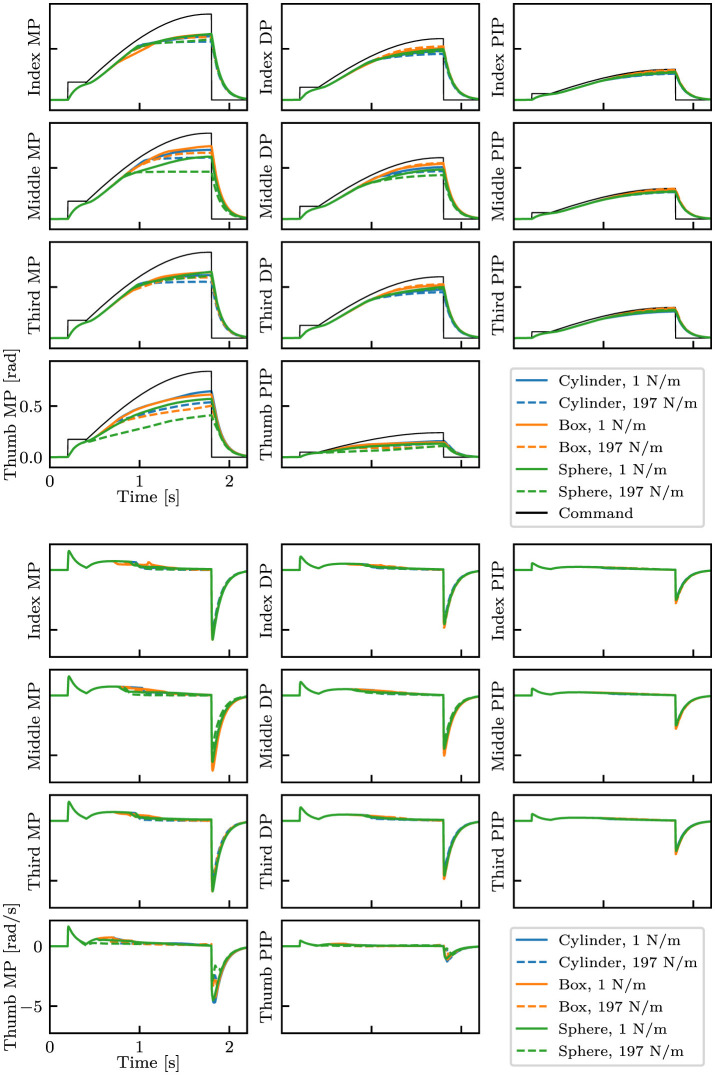
Examples of collected data with different shapes and stiffness. The top eleven plots show the finger joint angles and the commands, whereas the bottom eleven plots show the angular velocity of the joints. Three types of objects with the smallest (1 N/m) and highest (197 N/m) stiffness are shown.

For data collection, we controlled the robotic hand with a PD positional controller for 2.2 s with predefined joint angle commands as described in Table 1. For each episode, we recorded a time series of 33-dimensional data that consists of joint angles, joint velocity, and joint angle commands. The observation was measured. The delay is set to 1 ms. Examples of data are shown in Figure 4.

**Table 1:** Profile of joint angle commands.

**Time *t***	**Joint angle commands**
0.0 – 0.2 s	All commands are set to zero.
0.2– 0.4 s	Constant commands with the following amplitude: 0.175 rad for the thumb MP joint, 0.125 rad for the thumb DP joint,0.05 rad for the thumb PIP joint, and 0.0625 rad for the other joints.
0.4 – 1.8 s	Sinusoidal commands with the following amplitude: 0.84 rad for the thumb MP joint, 0.6 rad for the thumb DP joint, 0.24 rad for the thumb PIP joint, and 0.3 rad for the other joints.
1.8 – 2.2 s	All commands are set to zero.

We collected data with the three types of objects, while varying the stiffness with the range from 1 to 197 N/m in 4 N/m increments and varying the position and the orientation in 10 random values in the range of ±5 mm and ±5 deg, respectively; 1,500 data were collected in total. We call this dataset the *standard* dataset; we use this dataset for training unless otherwise specified. Seventy percentage of the dataset was used for training, and the remaining 30% were used for validation. During the course of training, we introduced Gaussian noises ε~N(ε;0,σ) to ***q*** and $\overset{\cdot }{q}$, while varying σ as $\log_{10}\sigma \sim U\left(\log_{10}\sigma ;-4,-1\right)$ for each mini-batch; here, U(x;a,b) denotes a uniform distribution of *x* within the range of *a* ≤ *x*<*b*.

In order to evaluate the model's ability to handle diverse data, we prepared another training dataset with varying object sizes and positions/orientations. In addition to the configuration of the standard dataset, objects of varying sizes are incorporated. This dataset comprises of 4,500 data points (1,500 data points each for the normal, bigger, and smaller sizes). We refer to it as a *full* dataset.

**Figure 5:**
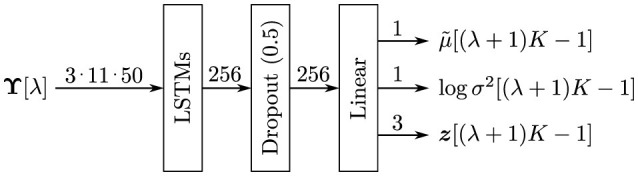
Neural network architecture.

We used a neural network architecture as illustrated in Figure 5. It consists of a recurrent layer with long short-term memories (LSTMs) with 256 units, followed by a dropout layer with a dropout rate of 0.5 and a full-connection layer. For learning stability, we calculate μ as follows:


(11) $$\begin{array}{l} \mu =\;200\cdot \frac{\tilde{\mu }+1}{2}, \end{array}$$

where $\tilde{\mu }$ denotes the output of a neural-network output. It should be noted that the above scaling is different from the scaling of loss function terms that keep the balance between *the gradient* of each term; the latter one is difficult to design. The hyperparameters utilized for training are listed in Table 2.

**Table 2:** Hyper-parameters for training.

**Item**	**Value**
Optimizer	Adam
Learning rate	10^−4^
Weight decay	10^−3^
#Epochs	50,000
Mini-batch size	64

## 3 Results

### 3.1 Estimation results with i.i.d. data

We first evaluated the proposed method with objects with stiffness that were unlearned but within the range of the training dataset. Precisely, the range of stiffness varied with the range from 3 to 195 N/m in 32 N/m increments. Additionally, the position/orientation was varied in 10 random values, resulting in a total of 210 data points. As these test data are almost independent and identically distributed (so-called i.i.d.) to the *standard* dataset, we will call them the *i.i.d*. dataset.

**Figure 6:**
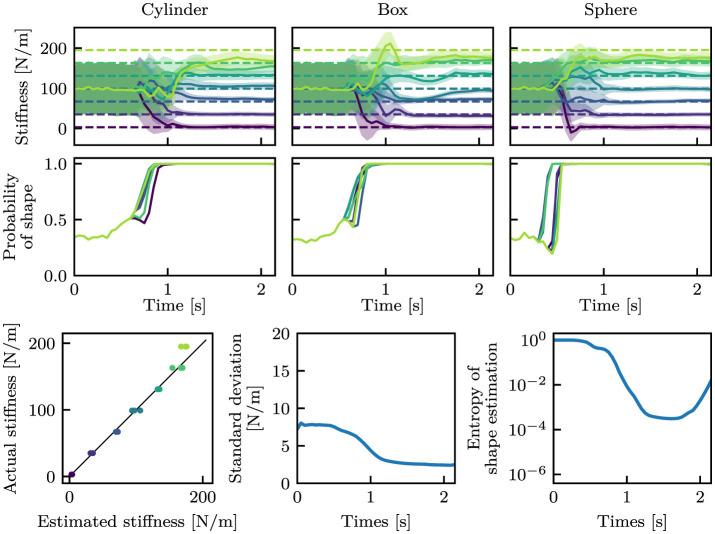
Estimation results with the i.i.d. dataset. The stiffness and shape estimation results are shown at the top and middle rows, respectively. For the stiffness estimation results, dashed lines indicate the true stiffness, and solid curves and filled areas indicate μ and σ. For the shape estimation results, only the estimated probabilities of the true shape classes are shown. The bottom-left graph shows the correspondence between the estimated mean stiffness at the final time step and the true stiffness, where the black line is *y* = *x*. The bottom-middle and bottom-right graphs show the mean standard deviations in stiffness estimation and the mean entropy in shape estimation, respectively.

Figure 6 shows the estimation results. Before touching the object (approximately before 0.5 s), the estimated mean stiffness was around 100 N/m with a large standard deviation in all cases. Additionally, the shape estimation was almost even (≈33%) for all classes. After touching the object, the estimated values converged close to near the true values. The standard deviations were also reduced, as the mean values converged to the true values. The entropy of shape estimation was also decreased a similar manner. At this juncture, the entropy *H* was calculated from the estimated probabilities of the shape as follows:


(12) $$\begin{array}{l} H≜-\overset{C}{\underset{c=1}{\sum }}p\left(s=S_{c}\right)\log_{C}p\left(s=S_{c}\right), \end{array}$$

where *p*(*s* = *S*_*c*_) indicates the estimated probability that the grasped object belongs to the class *S*_*c*_.

**Figure 7:**
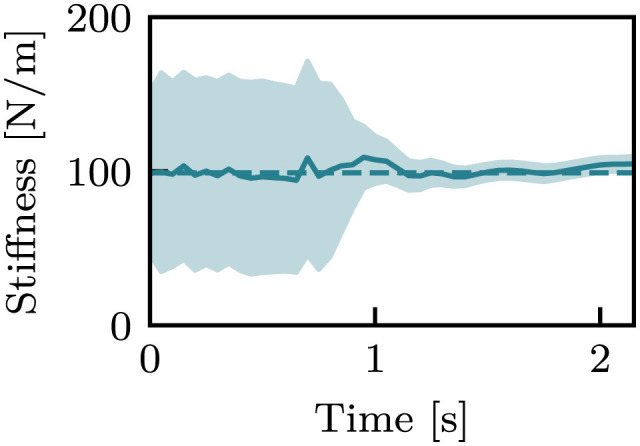
An estimation result with *k* = 99 N/m of the box-shaped object from the i.i.d. dataset. Dashed lines indicate the true stiffness, and solid curves and filled areas indicate μ and σ.

Figure 7 picks up the case of *k* = 99 N/m of the box-shaped object among Figure 6. The mean values were nearly constant throughout the episode, whereas the standard deviation drastically changed before and after the hand touched the object.

**Figure 8:**
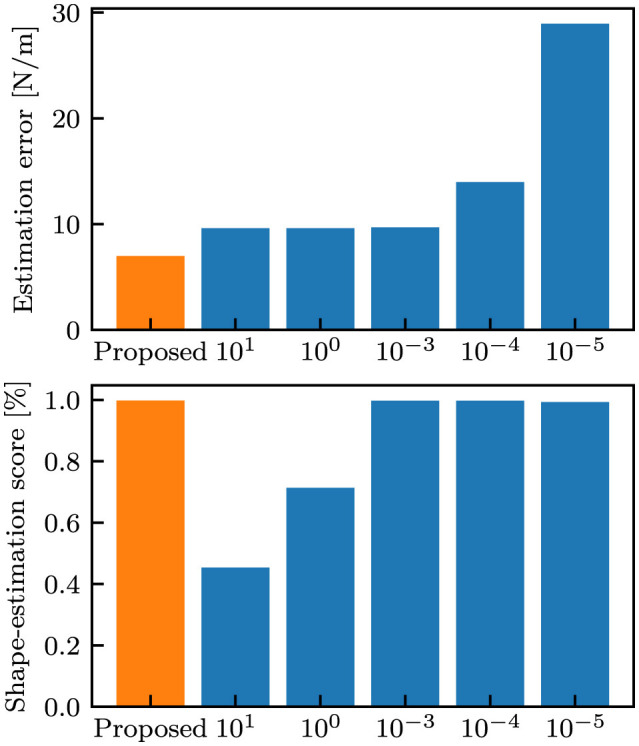
Comparison with baseline models. Each bar indicates the mean of the values at the final time step when evaluated on the *i.i.d*. dataset. The orange bars correspond to the proposed model, and the blue bars correspond to the baseline models, where the label values indicate α.

We also compared the proposed model trained with the *standard* dataset with baseline models that did not generate the stiffness variance. The baseline models were trained using the identical training dataset and identical hyper-parameters, with the exception of the following loss function:


(13) $$\begin{array}{l} L_{\text{baseline}}=\frac{1}{N}\overset{N-1}{\underset{n=0}{\sum }}\left[\alpha {\left(k_{n}-\mu_{n}\right)}^{2}+\text{CE}\left(s_{n},z_{n}\right)\right], \end{array}$$

where α indicates the weight coefficient of the stiffness errors. Figure 8 shows the performance comparison between the proposed model and baseline models with varying performance of α. In the baseline models, a low α resulted in large errors in stiffness estimation, whereas a high α resulted in low performance in shape estimation. The proposed model achieved the highest estimate estimation performance compared to the baseline models.

### 3.2 Estimation results with bigger objects

To evaluate how neural networks respond to novel objects, we evaluated the proposed method with objects with larger sizes than those in the training dataset. We shall refer to them as the *bigger* dataset.

**Figure 9:**
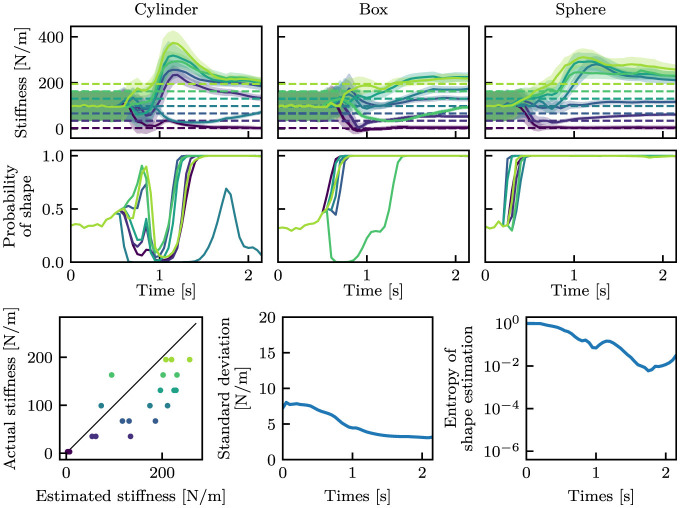
Estimation results with bigger objects. The stiffness and shape estimation results are shown at the top and middle rows, respectively. For the stiffness estimation results, dashed lines indicate the true stiffness, and solid curves and filled areas indicate μ and σ. For the shape estimation results, only estimates of the true shape classes are shown. The bottom-left graph shows the correlation between the estimated mean stiffness at the final time step and the actual stiffness, wherein the black line is *y* = *x*. The bottom-middle and bottom-right graphs show the mean standard deviations for stiffness estimation and the mean entropy for shape estimation, respectively.

Figure 9 shows the estimation results. It can be observed that the estimation errors were larger than those in Figure 6, i.e., the *i.i.d*. dataset. Also, the estimated values were largely fluctuated at the beginning of grasping (around 1.0 s).

**Figure 10:**
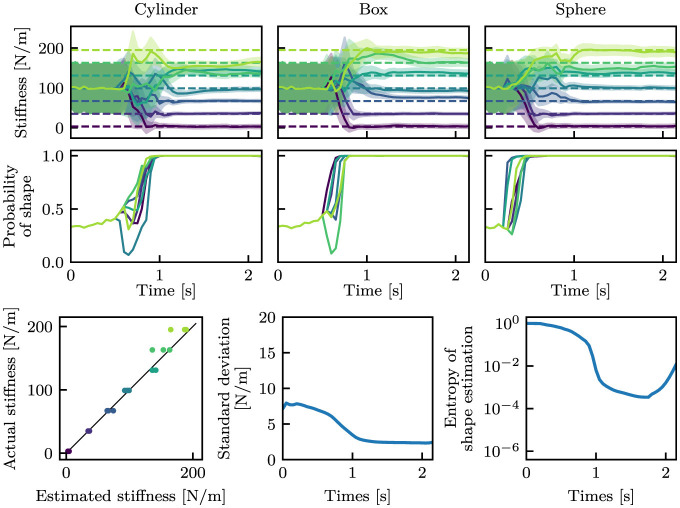
Estimation results with bigger objects by the proposed model trained with the *full* dataset. The stiffness and shape estimation results are shown at the top and middle rows, respectively. For the stiffness estimation results, dashed lines indicates the true stiffness, and solid curves and filled areas indicate μ and σ. For the shape estimation outcomes, solely the estimated probabilities of the true shape classes are shown. The bottom-left graph shows the correspondence between the estimated mean stiffness at the final time step and the true stiffness, where the black line is *y* = *x*. The bottom-middle and bottom-right graphs show the mean standard deviations in stiffness estimation and the mean entropy in shape estimation, respectively.

In order to demonstrate the capability of our method for more diverse data, we also trained the same architecture using the *full* dataset, which contains objects with varying sizes. Please note that training on a *full* dataset brings the *bigger* dataset within the learned range. Figure 10 shows the estimation outcomes. Compared to Figure 9, the model trained with the *full* dataset correctly estimated the object's properties.

### 3.3 Estimation results under observation noises

Subsequently, we assessed the efficacy of the proposed methodology in estimating object properties in the presence of observation noise. In this case, the setup other than observation noises was the same as in Section 3.1. We added two datasets with different observation noises to each dataset. One is achieved with the following noise applied:


(14) $$\begin{array}{ll} \varepsilon_{q} & \sim N\left(\varepsilon_{q};0\text{ rad},2\times 10^{-4}\;{\left(\text{rad}\right)}^{2}\right), \end{array}$$

(15) $$\begin{array}{ll} \varepsilon_{\overset{\cdot }{q}} & \sim N\left(\varepsilon_{\overset{\cdot }{q}};0\text{ rad}/\text{s},2\times 10^{-4}\;{\left(\text{rad}/\text{s}\right)}^{2}\right). \end{array}$$

This dataset is often referred to as the *small noise* dataset. Here, ε_*q*_ and $\varepsilon_{\overset{\cdot }{q}}$ denote the noise applied to the joint angles ***q*** and the joint angular velocity $\overset{\cdot }{q}$, respectively. Noises were incorporated for each time step and each degree of freedom autonomously. Another one has the following noise applied:


(16) $$\begin{array}{ll} \varepsilon_{q} & \sim N\left(\varepsilon_{q};0\text{ rad},8\times 10^{-4}\;{\left(\text{rad}\right)}^{2}\right), \end{array}$$

(17) $$\begin{array}{ll} \varepsilon_{\overset{\cdot }{q}} & \sim N\left(\varepsilon_{\overset{\cdot }{q}};0\text{ rad}/\text{s},8\times 10^{-4}\;{\left(\text{rad}/\text{s}\right)}^{2}\right). \end{array}$$

This dataset is often referred to as the *large noise* dataset.

**Figure 11:**
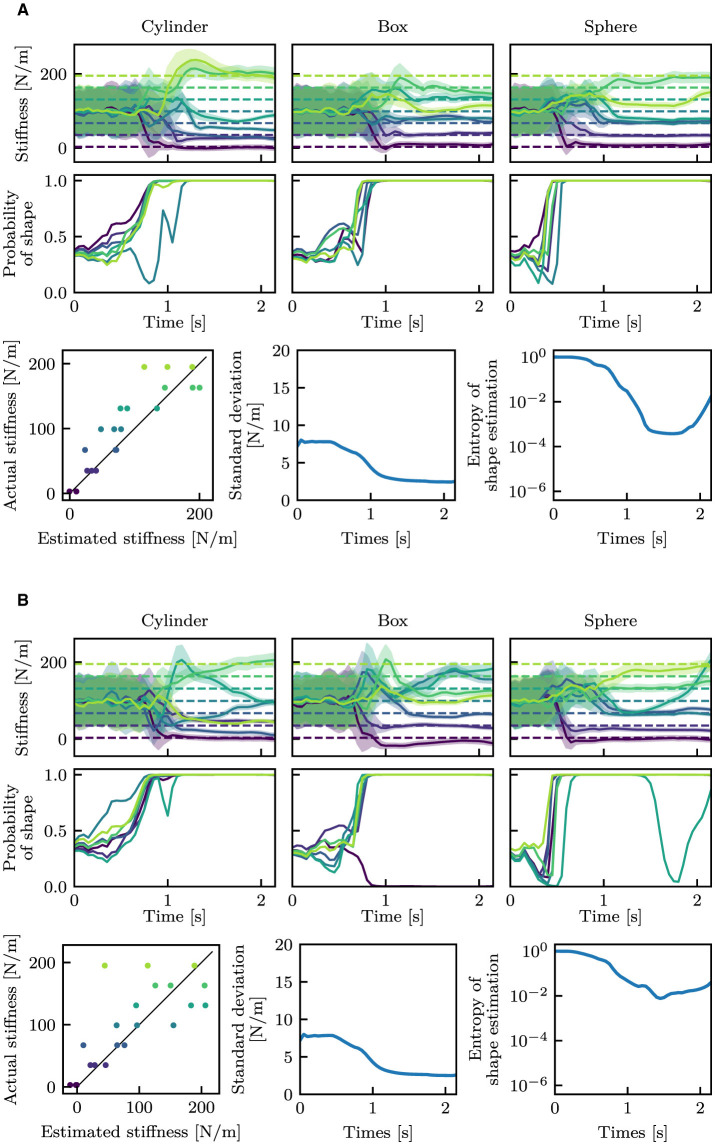
Estimation results with observation noises. The stiffness and shape estimation results are shown at the top and middle rows, respectively. For the stiffness estimation outcomes, dashed lines indicate the true stiffness, and solid curves and filled areas indicate μ and σ. For the shape estimation results, only the estimated probabilities of the true shape classes are shown. The bottom-left graph shows the correlation between the estimated mean stiffness at the final time step and the true stiffness, where the black line is *y* = *x*. The bottom-middle and bottom-right graphs show the mean standard deviations for stiffness estimation and the mean entropy for shape estimation, respectively. **(A)** Small noise. **(B)** Large noise.

Figure 11 shows the estimation outcomes. In contrast to Figure 6, the estimated values showed significant variation. Additionally, it can be observed that the estimated values fluctuated even before touch (before around 0.5 s).

### 3.4 Comparison of estimated variance

In the previous subsections, we tested trained neural networks with four test datasets: the *i.i.d., bigger, smaller*, and *large noise* datasets. In this section, we further analyze the estimated variance (standard deviation).

**Figure 12:**
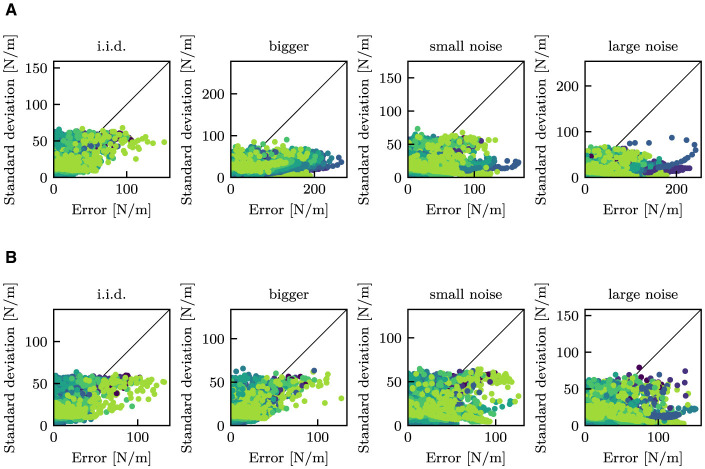
Comparison between errors and standard deviations. Here, the errors are the mean deviations between the true stiffness *k* and the estimated mean stiffness μ. Each point indicates an estimated value at each time step. Note that values after 0.75 s were shown to exclude values before touch. Colors correspond to the true stiffness; same as those in Figures 6–11. The black lines indicate that *y* = *x*. **(A)** The proposed model trained with the *standard* dataset. **(B)** The proposed model trained with the *full* dataset.

Figure 12 shows the relationship between the estimated standard deviation and the estimation errors for stiffness. It can be observed that the model trained on the *standard* dataset predicted significant high standard deviations when significant errors were detected in the *i.i.d*. dataset. In contrast, the model trained on the *full* dataset exhibited similar behavior on the *bigger* dataset in addition to the *i.i.d*. dataset. The correlation coefficients between the standard deviation and the errors are described in Table 3.

**Table 3:** Correlation coefficient between the standard deviation and the errors.

**Training dataset**	**I.i.d**.	**Bigger**	**Small noise**	**Large noise**
The *standard* dataset	0.659	0.322	0.308	0.176
The *full* dataset	0.712	0.661	0.457	0.286

**Figure 13:**
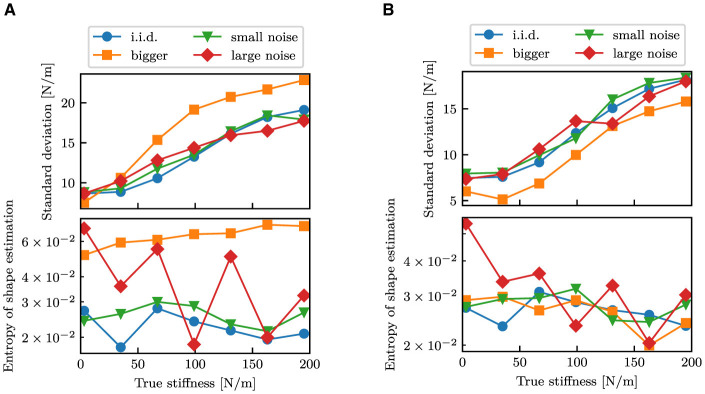
Comparison of the magnitude of estimation uncertainty between different test datasets. Each point indicates the mean standard deviation or the mean entropy after 0.75 s. **(A)** The proposed model trained with the *standard* dataset. **(B)** The proposed model trained with the *full* dataset.

Figure 13 shows variations in the estimated standard deviation of stiffness estimation and the entropy of shape estimation in relation to the authentic stiffness for the test datasets. The standard deviations generally increased with increasing stiffness, whereas the entropy decreased with increasing stiffness. For the *bigger* dataset, the proposed model trained with the *standard* dataset produced higher standard deviations and entropy compared to other datasets. In contrast, the proposed model was trained with the *full* dataset, which contains data with bigger objects, and resulted in compatible entropy and even lower standard deviations than the *i.i.d*. dataset.

## 4 Discussion

### 4.1 Discussions and conclusions

The proposed model was trained on the *standard* dataset and was able to estimate the stiffness and the shape from proprioception signals, i.e., joint angle and joint angular velocity. As shown in Figure 6, the trained neural network was capable of accurately estimating object properties from the *i.i.d*. dataset. In the other datasets, although the accuracy decreased, the stiffness was generally estimated using the correct large and small relationships. Furthermore, the shape was accurately estimated in the majority of instances. The capability of the model can be improved by using a more diverse dataset, as shown in Figure 9. The proposed model that was trained with the *full* dataset was able to perform well in the *bigger* dataset.

One of the primary objectives of this study is to generate the variance of stiffness estimation. The estimated variance can be regarded as the confidence level of estimation, including aleatoric and epistemic uncertainty. Variance as epistemic uncertainty can be clearly observed from Figure 7. In that result, the mean stiffness μ was almost constant throughout the episode. However, the standard deviation remarkably changed before and after touching the object. Before touch, the robot cannot estimate the stiffness, as it lacks information about the stiffness. This results in a high variance, which means high epistemic uncertainty. After touch, a wealth of information is provided. The robot is provided with information about the physical properties of the object is provided to the robot through proprioception signals, which allow the robot to decrease the variance. This fundamental change can be found in all cases in Figures 6–11. Moreover, as observed in Figure 13A, the variance was estimated to be larger for the *bigger, small noise*, and *large noise* datasets in comparison to the *i.i.d*. dataset. In contrast, in the model trained on the *full* dataset, the variance in the *bigger* dataset decreased (Figure 13B). This implies that the variance that could be reduced by incorporating additional training data appears to be a reflection of the degree of epistemic uncertainty. Based on these factors, it we can be inferred that the proposed models can explicitly indicate their level of confidence in the estimated values in the form of variance. This ability is very helpful when the robot will use the estimated values for motion planning and decision making.

The entropy of shape estimation displayed a resemblance to that observed in the standard deviations of stiffness. The entropy was high prior to contact, indicating a high degree of epistemic uncertainty. Also, the *large noise* dataset resulted in higher entropy than those in the *small noise* dataset, which would mean aleatoric uncertainty. Similar to stiffness estimation, the disparity in the *bigger* dataset between the two models can also be observed in shape estimation. The entropy of the *bigger* dataset was higher in the model trained on the *standard* dataset, whereas it was similar to the other test datasets in the model trained on the *full* dataset. Therefore, the models provide a confidence level for shape estimation based on uncertainty as the entropy, which would also help subsequent decision making.

A comparison between the proposed and baseline models suggests the superiority of the proposed methodology. As shown in Figure 8, the baseline models resulted in varying performance based on the weight coefficient, α. A low α resulted in a low performance in stiffness estimation, whereas a high α resulted in low performance in shape estimation. Therefore, the baseline model necessitates the appropriate value of α to attain satisfactory performance. It will be more difficult as the number of variables to be estimated increases since the number of weight coefficients to be adjusted also increases. In contrast, the proposed model performed the best both in stiffness and shape estimation without any weight coefficient to be adjusted. The balance between the two estimation tasks is automatically taken by the estimation of stiffness variance.

As shown in Figure 13, the estimated variance increased and the entropy slightly decreased with the increasing stiffness. The first reason would be due to the fact that the robot estimates the stiffness based on the deformation of the object through proprioception. According to Hooke's law, the stiffness *k* can be computed from the displacement δ*x* and contact force *f* in the following manner:


(18) $$\begin{array}{l} k=\frac{f}{\text{δ}x}. \end{array}$$

From the above relationship, it is clear that the amount of displacement is inversely proportional to the increase in stiffness. Therefore, in a high-stiffness object, it is imperative for the robot needs to assess its stiffness based on minimal displacements. This task is comparatively more challenging than estimating an object with low-stiffness. The reduction in variance for the *bigger* dataset in Figure 13B is explained by the fact that larger objects deform more significantly for the same stiffness, resulting in an easier task. On the other hand, the diminution in entropy may be attributed to the ease with which soft objects can deform. The large deformation will it difficult for the robotic hand to determine the original shape from finger joint angles. In the rigid objects, in contrast, the fingers can bend to follow the object's shape, resulting in an easier shape estimation. Results in Figure 13 may reflect those facts, suggesting that the neural networks could also estimate the confidence level according to the difficulty of the task.

Future work is to combine this strategy with control. Our method, neural networks can represent the confidence level of estimation, including uncertainty and task difficulty, as well as variance and entropy. This aids a task planner in determining the balance between exploration and exploitation. Recent studies have focused on approaches for modeling decision making through probabilistic inference, such as *control as inference* (Levine, 2018) and *active inference* (Friston et al., 2016). Our method, which is based on probabilistic inference, can be combined with other methods into a single probabilistic inference.

### 4.2 Related works

The measurement and estimation of stiffness holds significant importance in engineering. Thus, stiffness measurements have been made in some area. For example, Wang et al. (2016) summarized stiffness measurement methods for train areas. Marter et al. (2018) measured the stiffness of polymer foams using dot markers on the objects. Hattori and Serpa (2015) estimated the normal stiffness of the objects using a neural network. For robotic grasping and manipulation, Kicki et al. (2019) estimated object stiffness by measuring the contact force and the finger distance with varying grasping force. Spiers et al. (2016) combined tactile and actuator signals to assess the stiffness and the posture of the objects. For stiffness measurement, those methods use both contact force and displacement information. This is intuitive, since the Hooke's law argues that the stiffness *k* is a ratio of the displacement δ*x* and contact force *f*, i.e., $k=\frac{f}{\text{δ}x}$. On the other hand, in fact, it is also possible to estimate stiffness without directly measuring of force. For example, a reaction force observer (Murakami et al., 1993; Ohnishi et al., 1996) facilitates the estimation of external force in the absence of force sensors. Coutinho and Cortesão (2014) proposed a method for stiffness estimation method based on two force observers with different stiffness candidates. Also, Bednarek et al. (2021) estimated the stiffness of the grasped objects using a soft gripper with internal measurement units (IMUs) through learning. These studies support the possibility of stiffness estimation from proprioception signals. However, this is yet to be fully evaluated in the context of multi-finger robotic hands.

The estimate of object shape by contact has been investigated. Tsujimura and Yabuta (1989) proposed a method for object-shape detection by using a probe with a six-axis force/torque sensor. Mimura and Funahashi (1994) classified tools based on their contact state, which can be identified by force/torque signals. Also, the force signal-based object-shape estimation is often realized using particle filters (von Drigalski et al., 2020; Kutsuzawa et al., 2020; Bimbo et al., 2022). Drimus et al. (2014) developed a novel approach. The tactile array sensor utilizes piezoresistive rubber and thread electrodes for object classification, coupled with a gripper. Gao et al. (2016) used convolutional neural networks to associate haptic and visual measurements with haptic adjectives, such as *rough, hairy*, and *soft*. There are also methods to estimate the object shape by using a particle filter (Behbahani et al., 2015) and a Gaussian process (Khadivar et al., 2023), both of which are based on probabilistic inference. According to those methods, probabilistic inference is effective for geometric-property estimation. Based on this fact, we also evaluate geometric properties using a probabilistic way unified with stiffness estimation in this study.

There are numerous robotic hands with tactile sensors (Dahiya et al., 2010; Narita et al., 2020; Cirillo et al., 2022; Spiers et al., 2016). Although tactile sensors and their applications are undergoing development, they are still in their infancy. First, high-precision tactile sensors are expensive; this is a problem when considering mass production and industrial applications. Second, sensors are often sensitive to external disturbances such as temperature, light, or electromagnetic fields. Given their cost and robustness against the external environment, the primary use of proprioception sensors is effective. Robotic hands can have the ability to handle contact even without tactile sensors. The robotic hand developed by Ajoudani et al. (2013) does not have force sensors, but instead estimates contact force by using interaction torque observers. Xu et al. (2021) developed a soft gripper with a grasping force estimation method using a neural network based on the amount of deformation captured by a camera. Nagabandi et al. (2020) and Andrychowicz et al. (2020) used a robotic hand with a 24 degrees-of-freedom robotic hand for dexterous manipulation with reinforcement learning.

Handling uncertainty is an important topic in machine learning field. Uncertainties are sometimes classified into *aleatoric* and *epistemic* uncertainty with the former referring to uncertainty due to randomness, and the latter referring to uncertainty caused by lack of knowledge (Hora, 1996; Hüllermeier and Waegeman, 2021). A common approach to uncertainty is probabilistic inference, such as the Gaussian process, Kalman filter, and Monte Carlo methods, but these methods have limited representation ability or are often computationally expensive in high-dimensional signal processing. For methods based on neural networks, Bayesian neural networks can naturally perform Bayesian inference (Denker and LeCun, 1990), but the naive way can also be computationally expensive. Nix and Weigend (1994) first proposed a neural network that could generate the mean and variance of the target-data distribution. This method is often used because it is simple to implement and calculate. As per recent well-known models, variational auto-encoders (Kingma and Welling, 2013) utilize neural networks to generate the mean and variance to represent a Gaussian distribution of latent variables. Deep reinforcement learning methods also incorporate policy neural networks that generate variance of actions (Haarnoja et al., 2018). More details can be figured out in Gawlikowski et al. (2023). As robotic applications of variance-output neural networks, Ding et al. (2021) developed neural networks model for soft actuators that predict the actuator's position, contact location, and contact force, including their variance. Also, Takahashi et al. (2021) used neural networks treats estimate estimation errors and treat the estimated estimation errors as the actual amount of epistemic uncertainty. We adopted the approach of Nix and Weigend (1994) for stiffness estimation due to its simplicity, while addressing the shape classification task together. We will demonstrate that this approach can not only can quantify estimation uncertainty, but also eliminate hyper-parameters from the training loss function.

## Data Availability

The datasets presented in this study can be found in online repositories. The names of the repository/repositories and accession number(s) can be found below: https://github.com/kyo-kutsuzawa/object_property_estimation_in_robotic_hand.
